# Supplementary material for: Biasing the conformation of ELMO2 reveals that myoblast fusion can be exploited to improve muscle regeneration
Source: Nat Commun. 2022 Nov 18;13:7077. doi: 10.1038/s41467-022-34806-4 (PMC9674853; doi:10.1038/s41467-022-34806-4)
Supplement: Supplementary file 1 — supplementary information [file 41467_2022_34806_MOESM1_ESM.pdf]

Tran et al.

Biasing the conformation of ELMO2 reveals that myoblast fusion can be exploited for regenerative therapy

Supplementary information

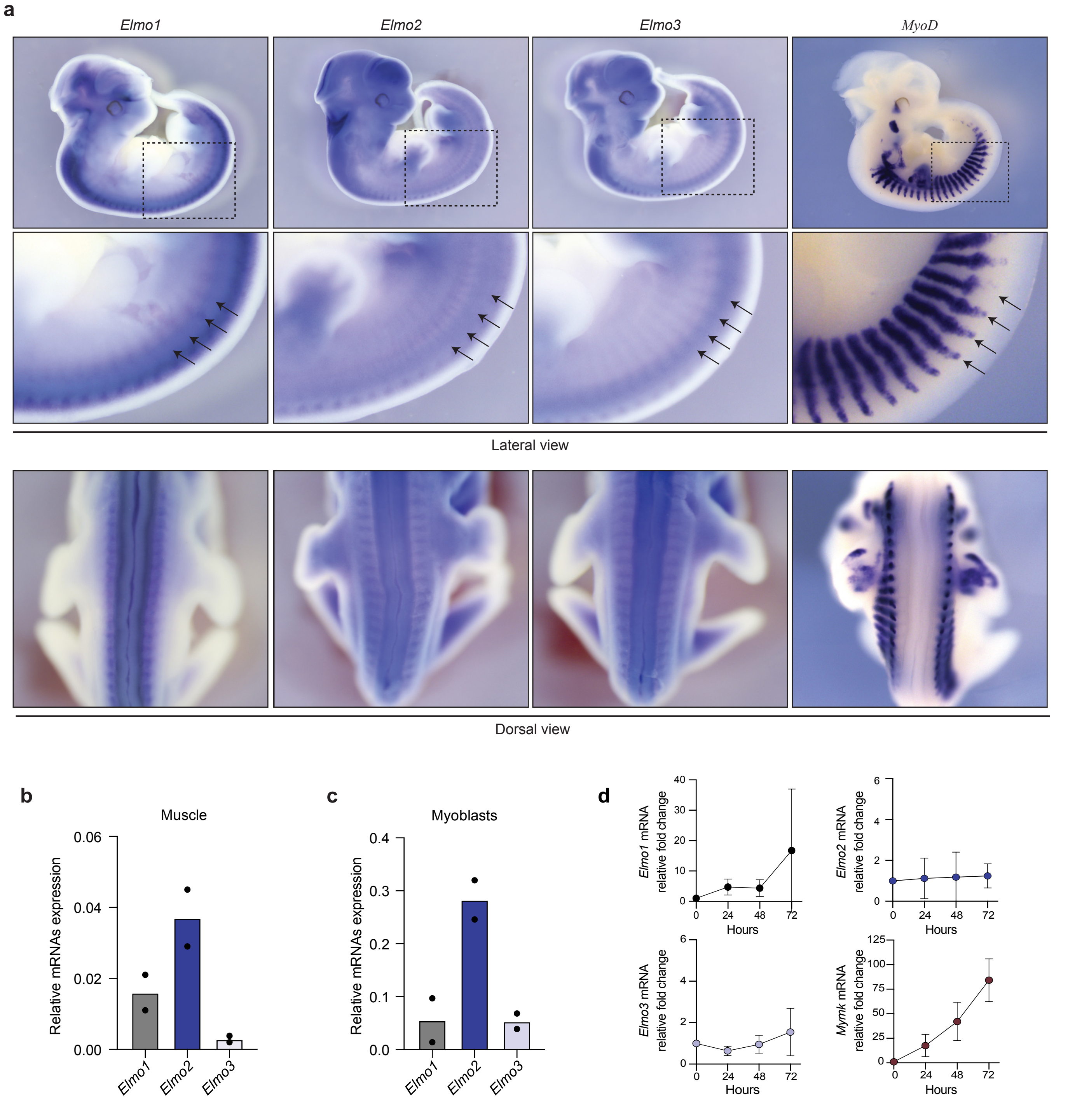

Supplementary Figure 1. *Elmo* mRNAs are expressed in skeletal muscle and muscle progenitors. a) Whole mount *in situ* hybridization using DIG-labelled anti-sense probes showing expression of *Elmo1*, *Elmo2*, *Elmo3* and *MyoD* mRNAs in E11.5 mouse embryos. *Elmo* mRNAs are broadly expressed during embryogenesis, including in somites (pointed by the arrows). **b-c**) qRT-PCR assays demonstrating the relative mRNAs expression of *Elmo1*, *Elmo2* and *Elmo3* (using the reference gene *mB2M*) in **b**) the TA muscle and **c**) primary myoblasts of WT mice. Bars are the mean of two different mice (dots). d) qRT-PCR assays demonstrating the relative change in mRNAs of *Elmo1*, *Elmo2* and *Elmo3* (using the reference gene b-actin) in C2C12 myoblasts from 0 to 72 h of differentiation. Data are the mean +/- SD of 3 independent experiments. Source data are provided as a Source Data file.

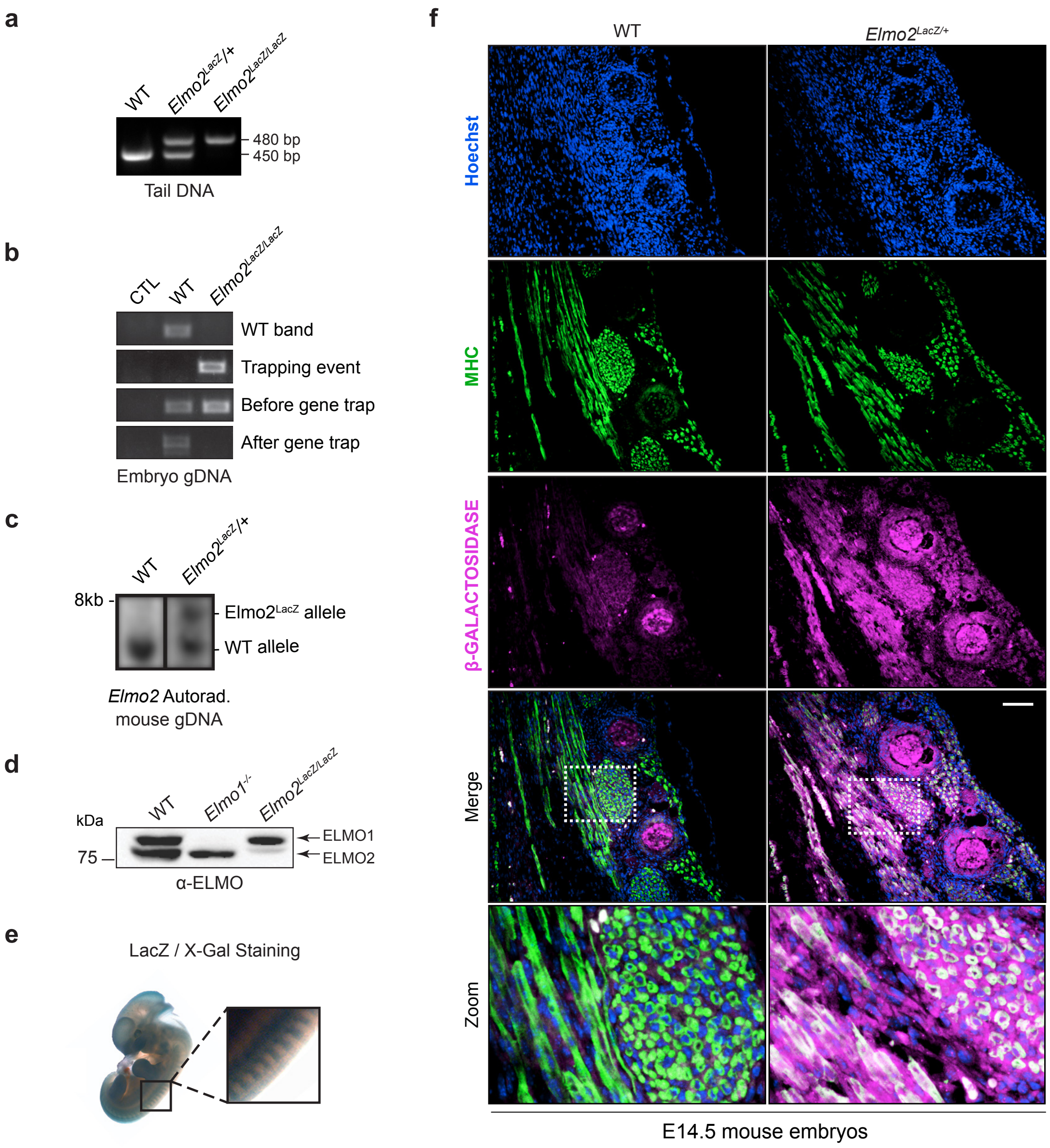

Supplementary Figure 2. Characterization of the *Elmo2*^{LacZ} mouse model. To validate the generation of the *Elmo*^{LacZ} mouse model, different approaches were used. **a**) PCR genotyping on mouse tail DNA yielded the following sizes of amplicon: 450 bp (WT) and 480 bp (*Elmo2*^{LacZ}). **b**) RT-PCR analyses using RNA isolated from embryos confirms the trapping event in the *Elmo2*^{LacZ} mice, thus demonstrating a functional LacZ reporter system. H_2O was used as control. **c**) Southern blot results on genomic DNA isolated from WT and *Elmo2*^{LacZ} heterozygous animals further confirms the proper rearrangement in the locus. The genomic DNA was digested with Eag1/BglII and probed with a 3' intronic *Elmo2* fragment amplified by a PCR reaction containing ^{32}P-ATP (see Fig. 1a). **d**) Western blot demonstrating the expression of both ELMO1 and ELMO2 in WT embryo and confirming the loss of expression of ELMO1 in *Elmo1*$^{-/-}$ embryo and of ELMO2 in *Elmo2*^{LacZ} embryo. Data are representative of 3 independent experiments. Source data are provided as a Source Data file. **e**) Whole mount X-gal staining of E11.5 heterozygous *Elmo2*^{LacZ} embryo demonstrates expression of ELMO2 during embryogenesis, including in somites. **f**) E14.5 embryo sections stained with an anti-β-GALACTOSIDASE antibody (magenta), demonstrating the expression of ELMO2 in muscle cells. Myofibers are stained with an anti-MHC antibody (green) and nuclei were revealed with Hoechst (blue). This experiment was performed on two embryos per genotype (Scale bar: 50 μm).

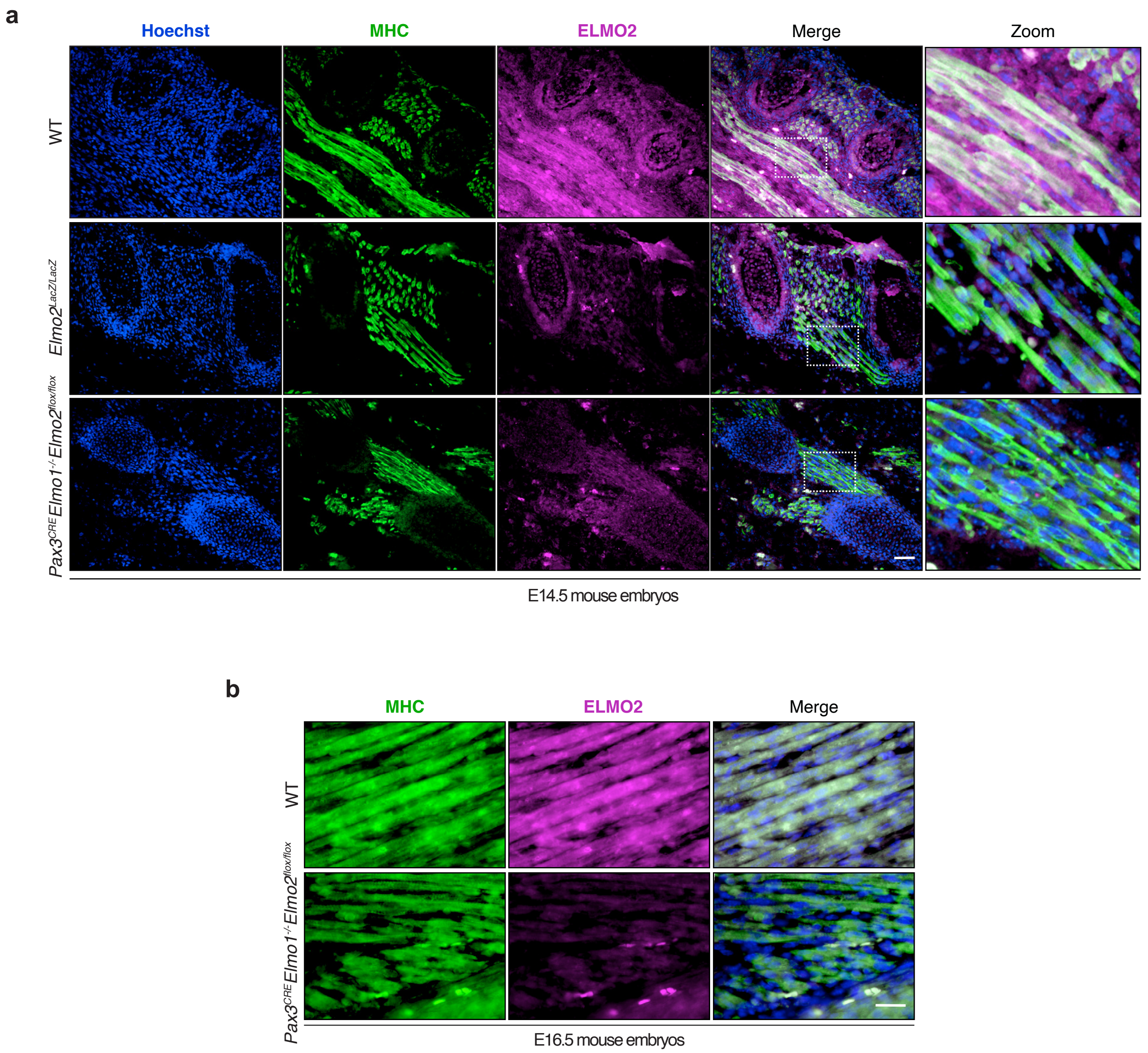

Supplementary Figure 3. ELMO2 expression during muscle development. Longitudinal sections of **a**) E14.5 and **b**) of E16.5 embryos of the indicated genotypes were stained with antibodies against ELMO (magenta) and MHC (green), and Hoechst (blue) was used to reveal the nuclei. These staining demonstrate the expression of ELMO2 in muscle cells. Notably, in (b), no signal is obtained for the anti-ELMO antibody in *Pax3^{CRE}Elmo1$^{-/-}$Elmo2$^{flox/flox}$* embryonic muscle, thus confirming the genetic ablation of *Elmo1* and *Elmo2*. Data are representative of 3 independent experiments. (Scale bar: a = 50 µm; b = 25 µm).

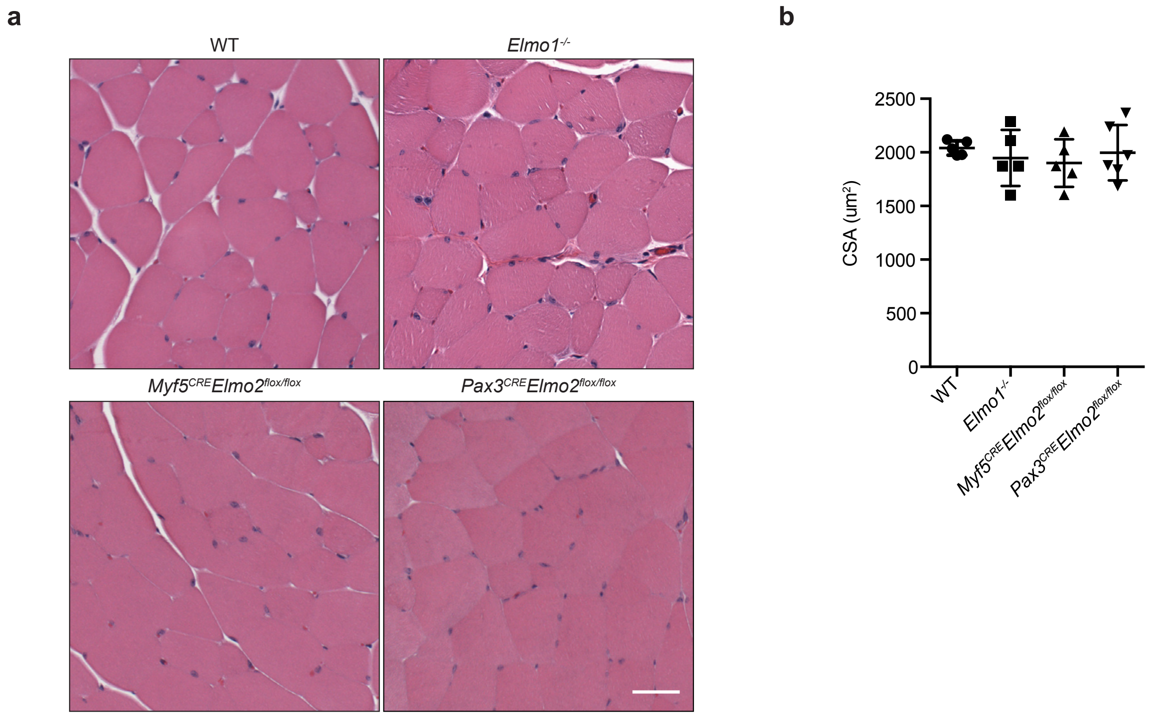

Supplementary Figure 4. ***Elmo1^KO^* and *Elmo2^cKO^* mice present normal muscle growth. a**) Representative muscle cross-sections of 3 months old mice of the indicated genotypes stained with H&E (n=5). Scale bar: 50 µm. **b**) Quantification of (a) where the CSA of the myofibers of the indicated mice was analyzed. Data are presented as mean values +/- SD (n=5).

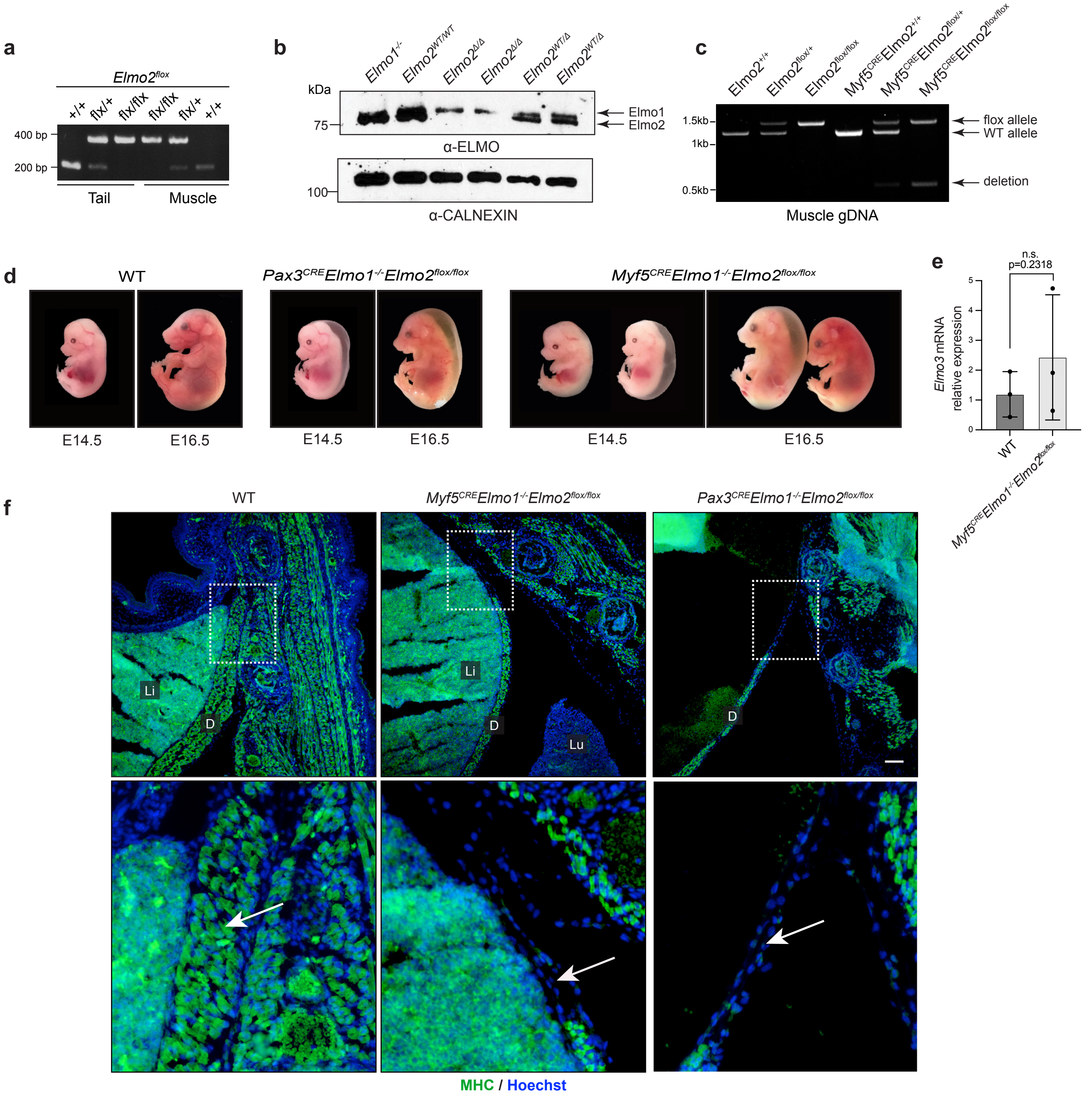

Supplementary Figure 5. Characterization of *Elmo2^{flox}* and *Elmo1$^{-/-}$Elmo2^{flox}* mice. a) Genotyping of *Elmo2^{flox}* mice. PCR analysis of the WT (+/+), heterozygous (flx/+) and homozygous (flx/flx) alleles on genomic DNA isolated from tail and muscle tissues. **b**) To confirm the genetic inactivation of *Elmo2* following the Cre/LoxP recombination, *Elmo2^{flox}* mice were interbred with a total Cre deleter mouse, i.e. *Meox2^{CRE}*. Western blot performed on total E9.5 embryo lysates confirmed the absence of ELMO2 expression in *Elmo2$^{\Delta/\Delta}$*mice. Data are representative of 3 independent experiments. **c**) PCR analyses performed on genomic DNA from muscle tissues of *Myf5^{CRE}Elmo2^{flox}* mice reveals the WT allele, the flox allele and the deletion following Cre-LoxP recombination, for the indicated genotypes. **d**) Representative pictures of embryos with the indicate genotypes isolated at E14.5 and E16.5. **e**) qPCR on limbs dissected from E14.5 aged embryos shows that *Elmo3* mRNA expression is unchanged in *Myf5^{CRE}Elmo1$^{-/-}$Elmo2$^{flox/flox}$* mice compared to WT mice. mRNA levels were normalized relative to actin mRNA expression. Graph represents mean +/- SD of 3 embryos per condition. The student's t test with welch's correction (unpaired) was used to calculate the P value. **f)** The muscle responsible for the attachment of the diaphragm with the ribs (pointed by the arrow) is missing in both *Myf5^{CRE}Elmo1$^{-/-}$Elmo2$^{flox/flox}$* and *Pax3^{CRE}Elmo1$^{-/-}$Elmo2$^{flox/flox}$* embryos. Li: liver; D: diaphragm; Lu: lung. Muscle cells were stained with an anti-MHC (green) antibody and Hoechst (blue) was used to reveal the nuclei. Experiments were done on two embryos per genotype. (Scale bar: 100 µm).

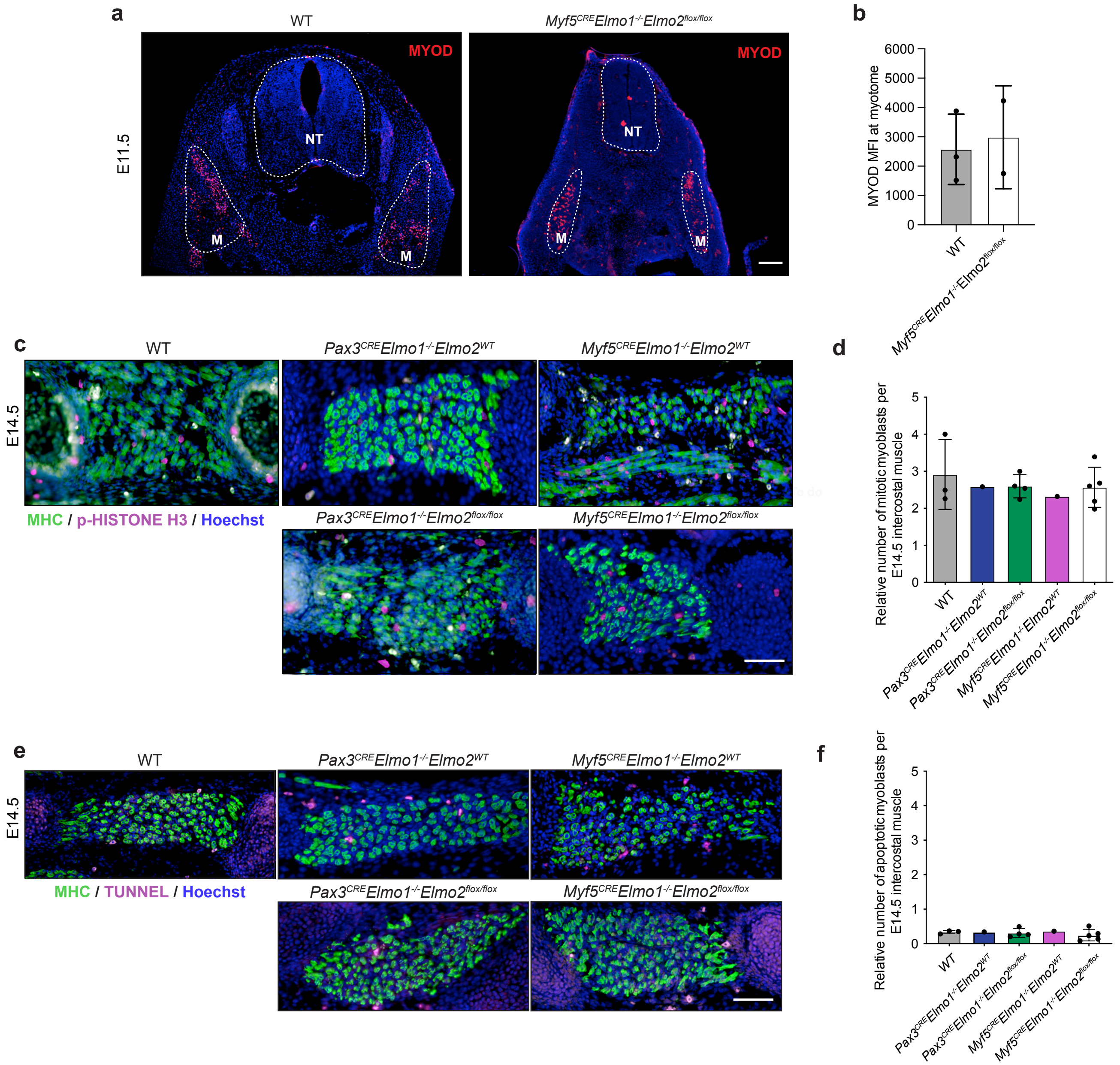

Supplementary Figure 6. Characterization of the differentiation, proliferative and apoptotic profiles of themuscle precursors in the absence of ELMO proteins. a) Cross-sections of E11.5 embryos of the indicatedgenotype. Cells undergo properly differentiation, as shown by the staining against MYOD (red), which isexpressed similarly in WT and $Myf5^{CRE}Elmo1^{-/-}Elmo2^{flox/flox}$ embryos. M: myotome; NT: neural tube. **b)** Quantification of the mean fluorescence intensity (MFI) +/- SD of the MYOD staining at the myotome (n = 3 embryos for WT and 2 embryos for $Myf5^{CRE}Elmo1^{-/-}Elmo2^{flox/flox}$). **c-f)** Proliferative and apoptotic profiles of muscle cells in E14.5 embryos. **c, e**) Representative longitudinal sections showing intercostal muscles of the indicated embryos. **c**) Sections stained with anti-MHC (green) and anti-phospho-HISTONE H3 (magenta) antibodies to reveal mitotic muscle cells. **d**) Data are the mean +/- SD of the number of mitotic cells found in (b) from multiple independent sections (n=3 WT embryos, n=1 $Pax3^{CRE}Elmo1^{-/-}Elmo2^{WT}$ or $Myf5^{CRE}Elmo1^{-/-}Elmo2^{WT}$embryos, n=4 $Pax3^{CRE}Elmo1^{-/-}Elmo2^{flox/flox}$, n=5 $Myf5^{CRE}Elmo1^{-/-}Elmo2^{flox/flox}$ embryos). (**e**) Sections stained with an anti-MHC (green) antibody and using TUNEL (magenta) assay to detect apoptosis. **f**) Data are the mean +/- SD of the number of apoptotic cells found in (d) from multiple independent sections(n=3 WT embryos, n=1 $Pax3^{CRE}Elmo1^{-/-}Elmo2^{WT}$ or $Myf5^{CRE}Elmo1^{-/-}Elmo2^{WT}$embryos, n=4 $Pax3^{CRE}Elmo1^{-/-}Elmo2^{flox/flox}$, n=5 $Myf5^{CRE}Elmo1^{-/-}Elmo2^{flox/flox}$ embryos). Nuclei are stained with Hoechst (blue). Magnification: a= 40X; c, e =20X. (Scale bar: a = 100 μm; c, e = 50 μm). Source data for b, d and f are provided as a Source Data file.

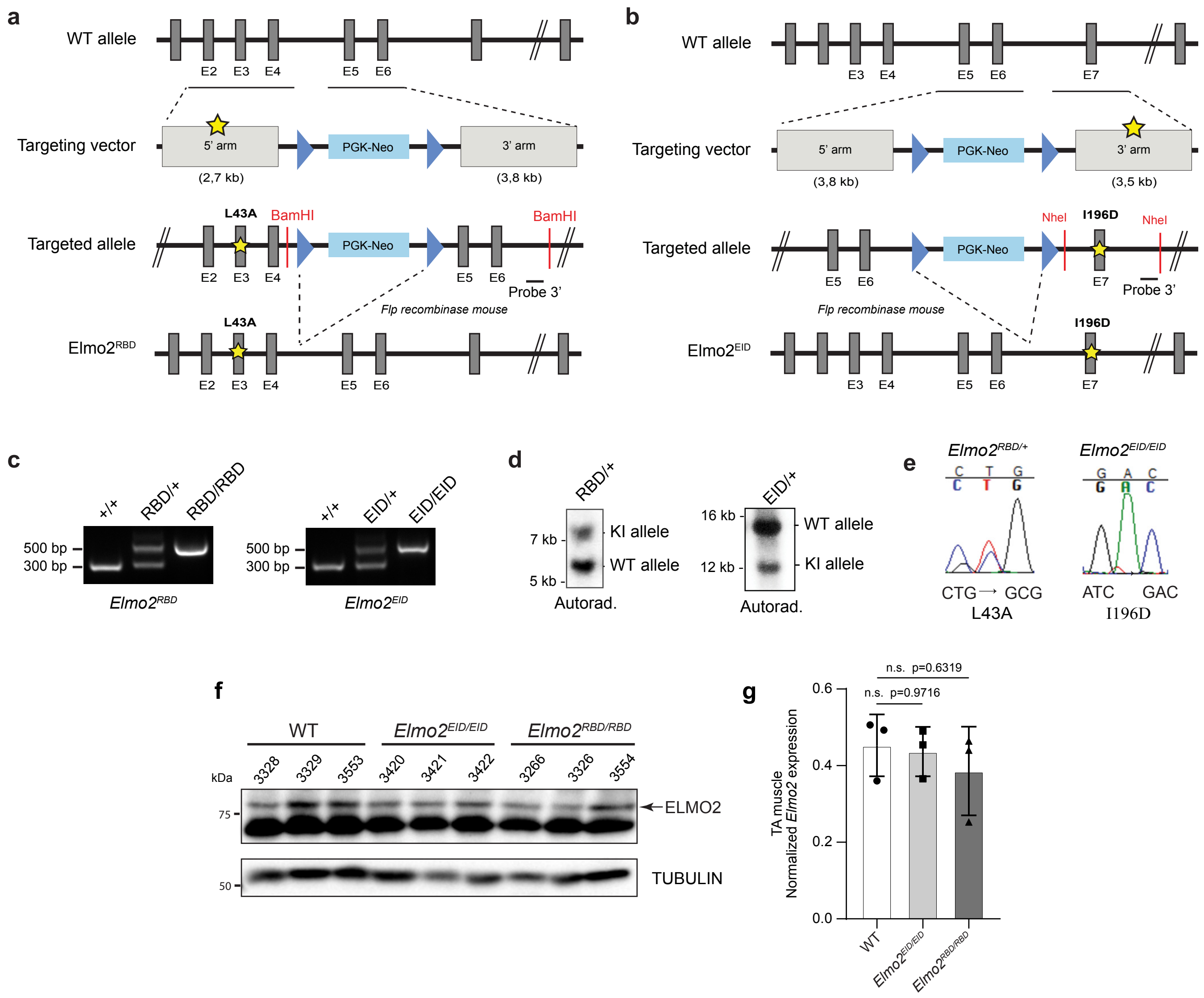

Supplementary Figure 7. *Elmo2* mutant mice with mutations regulating the conformation of the ELMO-DOCK complex. a, b) Partial representation of the *Elmo2* locus to demonstrate the strategy of homologous recombination for the generation of **a**) *Elmo2^{RBD}* (L43 mutation) and b) *Elmo2^{EID}* (I196D mutation) mice. The WT alleles, the targeting vectors and the targeted alleles are illustrated. The southern blot strategy is demonstrated (digestion enzymes and probes used). **c**) Genotyping analyses for the indicated genotypes. **d**) Southern blot analysis using tail DNA isolated from the indicated genotypes confirmed the proper rearrangements in the *Elmo2* locus. The genomic DNA was digested with BamHI for *Elmo2^{RBD}* and NheI for *Elmo2^{EID}*, and analyzed with an intronic *Elmo* probe in 3' amplified by PCR from the genomic DNA in the presence of ^{32}P-ATP. Data are representative of 3 independent experiments. **e**) Sequencing results demonstrating the indicated mutations, confirming the presence of L43A mutation in the RBD and I196D mutation in the EID of *Elmo2*. **f)** TA muscles from 3 different mice were dissected and lysated. Western blots using myosin heavy chain (MHC), ELMO2 and TUBULIN antibodies shows that ELMO2 expression is not significantly different in *Elmo2$^{EID/EID}$* and *Elmo2$^{RBD/RBD}$* mice as compared to WT mice. **g)** ELMO2 protein level was normalized relative to TUBULIN protein level. Graph represents the mean +/- SD of the 3 mice per condition. P-values were calculated using one-way ANOVA and were adjusted using the turkey's multiple comparisons test. Source data are provided as a Source Data file.

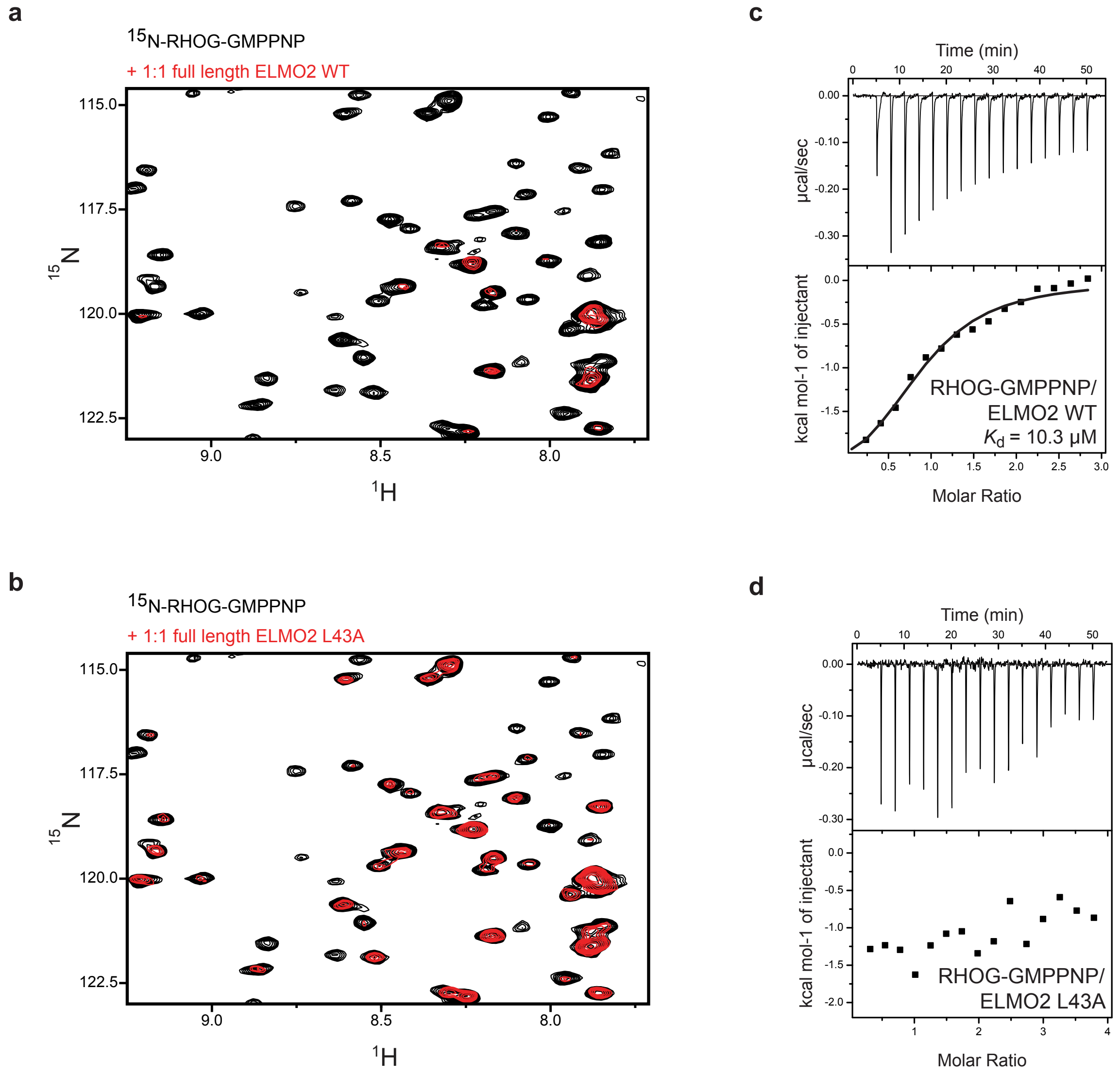

Supplementary Figure 8. Biochemical characterization of ELMO2^{WT} and ELMO2^{RBD} (L43A) for their interactions with the small GTPase RHOG. a) Overlay of ^{1}H-^{15}N HSQC spectra of ^{15}N-labeled GMP-PNP-loaded RHOG (100μM) in the absence (black) and presence (red) of Elmo2 WT full-length (50μM). **b**) Overlay of ^{1}H-^{15}N HSQCs of ^{15}N-labeled, GMPPNP-loaded RHOG (100μM) in the absence (black) and presence (red) of ELMO2 L43A full-length (50μM). **c**) ITC thermogram for ELMO2 full-length WT titrated to RHOG-GMPPNP. **d**) ITC thermogram for ELMO2 full-length L43A titrated to RHOG-GMPPNP.

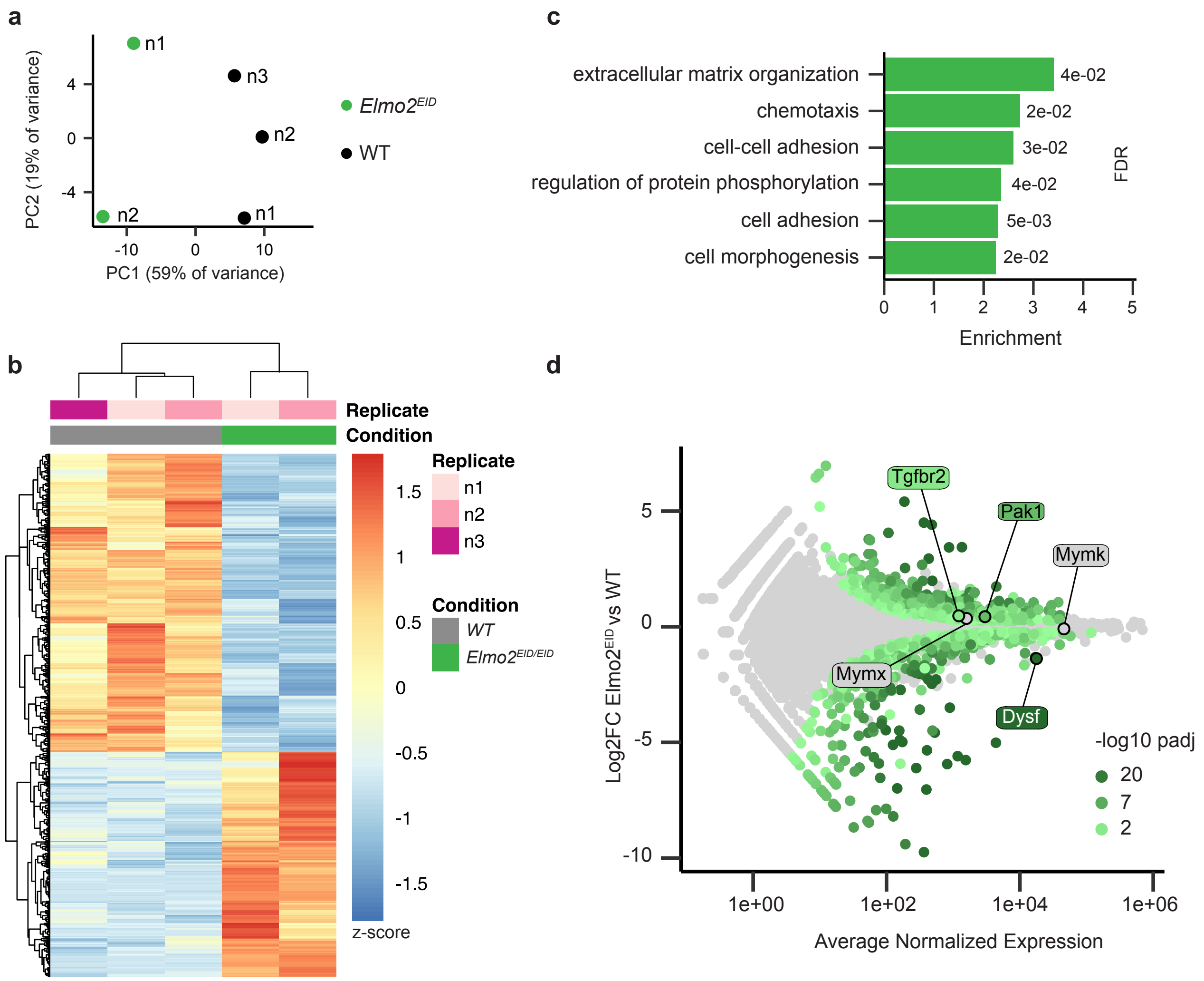

Supplementary figure 9. RNAseq analysis reveales a subset of genes differentiallly expressed between WT and *Elmo2*$^{EID/EID}$ primary mouse myoblasts. a) Principle component (PC) analysis of transcriptomes from differentiated myoblasts of WT and *Elmo2*$^{EID/EID}$ mice. **b)** Heatmap of the 961 differentially expressed genes (DEG; padj<0.05, Wald Test *Elmo2*$^{EID/EID}$ vs WT adjusted for multiple comparisons using the Benjamini-Hochberg method) between WT and *Elmo2*$^{EID/EID}$ myoblasts. Samples and genes are separated using Euclidian distance. **c)** Enrichment of select gene ontology (GO) terms associated with DEGs which are statistically overrepresented (FDR<0.001) when compared to all expressed genes. **d)** MA Plot of all expressed genes. Statistically significant DEGs are colored in green, and select genes involved in myoblast fusion are highlighted.

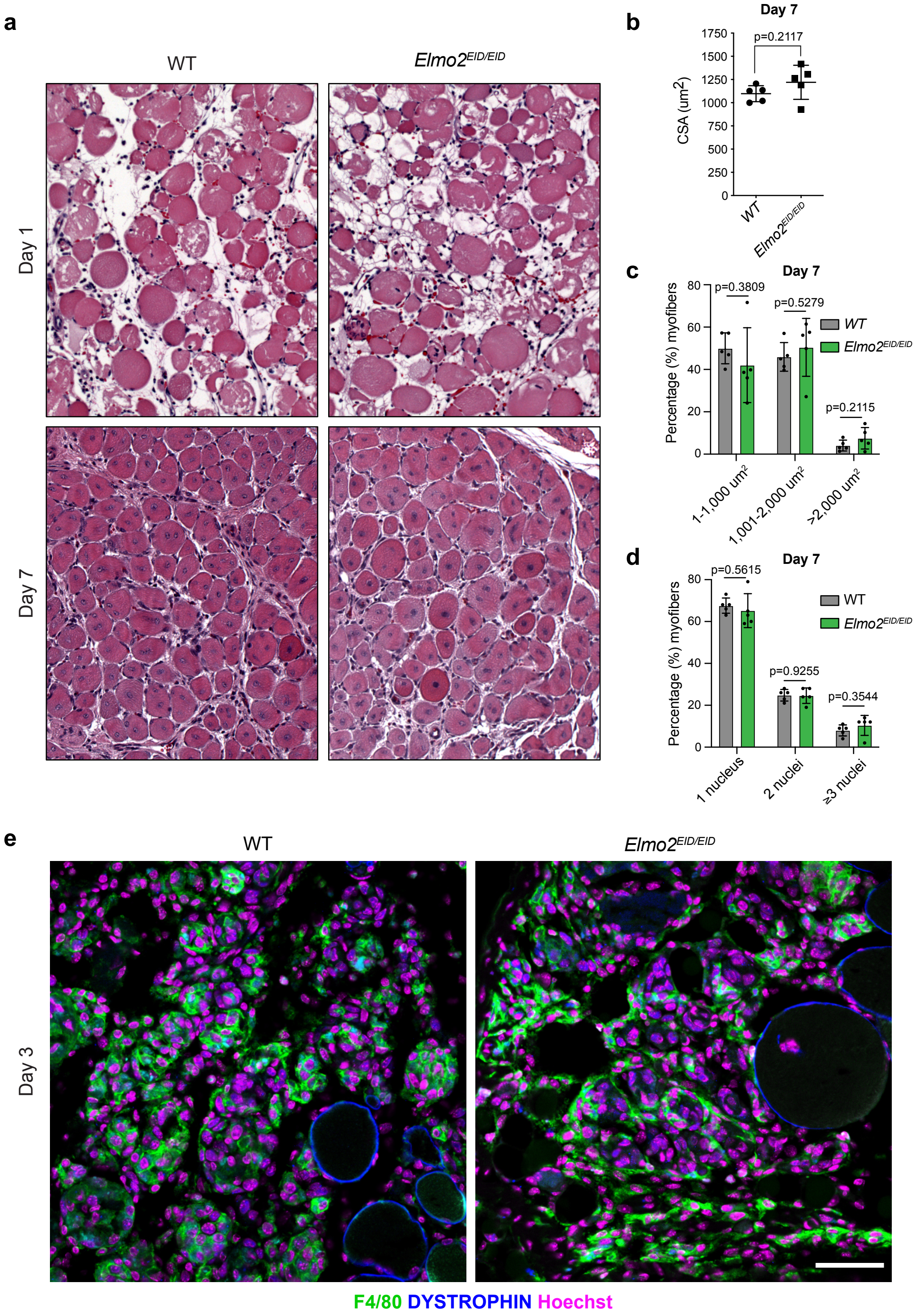

Supplementary Figure 10. Impact of ELMO2 conformational regulation on myoblast fusion early after CTX-induced injury. a) Representative cross-sections of TA muscle 1 or 7 days following CTX-induced injury. The graphs show the mean +/- standard deviation of b) the mean CSA per mice, c) the percentage (%) of myofibers at different ranges of myofiber size or d) number of nuclei per myofiber at 7 days following CTX-induced injury (n = 5 mice). The Student's t test (for comparison of two independent groups) was used to calculate the two-tailed P values. Source data are provided as a Source Data file. e) Infiltration of F4/80-positive macrophages (in green) 3 days following CTX-induced injury is not affected in *Elmo2$^{EID/EID}$* mice. The basement membrane of muscle was stained with anti-DYSTROPHIN (blue) and nuclei were stained with Hoechst (magenta). Scale bar: 50µm. Results are representative of 3 injured mice per condition.

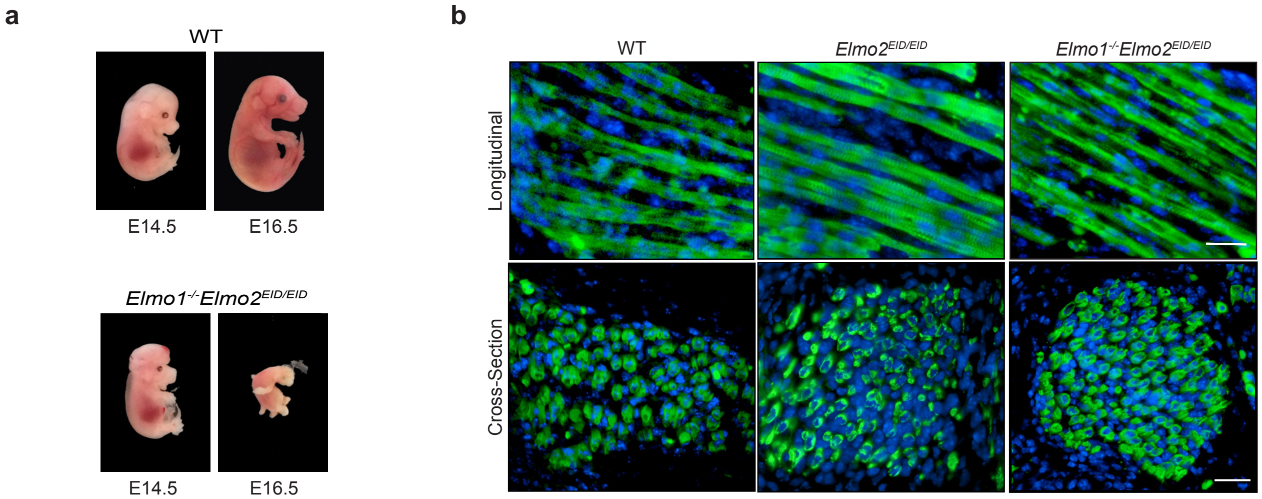

Supplementary Figure 11. *Elmo1$^{-/-}$Elmo2$^{EID/EID}$* mice developed multinucleated myofibers. a) Representative pictures of WT and *Elmo1$^{-/-}$Elmo2$^{EID/EID}$* mouse embryos dissected at E14.5 and E16.5. **b**) Longitudinal and cross-sections of E14.5 embryos. Muscle fibers stained with anti-MHC antibody (green) are present in WT, *Elmo2$^{EID/EID}$* and *Elmo1$^{-/-}$Elmo2$^{EID/EID}$* embryos. Nuclei are revealed with Hoechst (blue). Experiment was done on 3 embryos per genotype. Magnification:20X (longitudinal) and 40X (cross-section). (Scale bar: 25 μm).

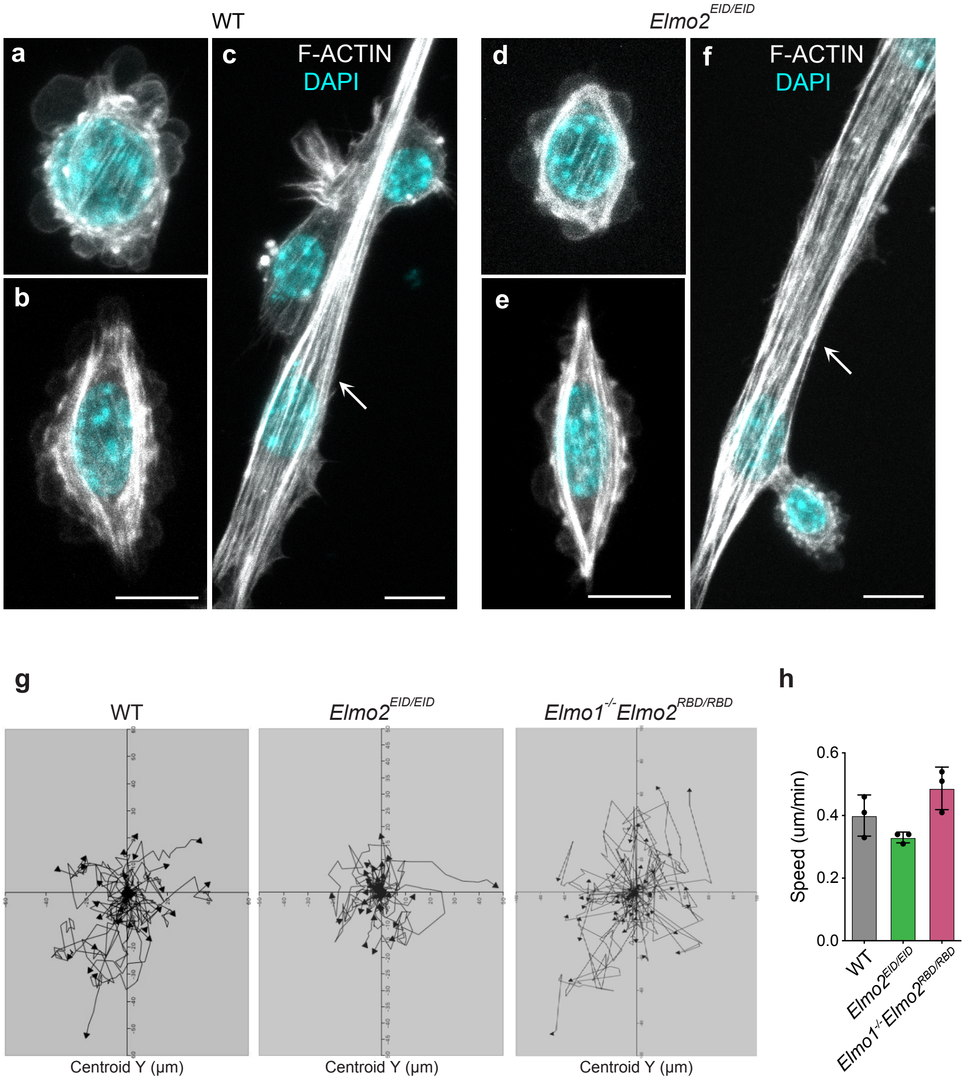

Supplementary Figure 12. Effect of ELMO2 conformation on myoblast F-ACTIN organization and migration. a-f) Primary myoblasts isolated from WT or *Elmo2*$^{EID/EID}$ mice were fixed after 24h in differentiation media and stained with ALEXA fluor-phalloidin (F-ACTIN) and DAPI (nucleus). Images show that the actin organization in different stages of differentiation were not affected by the ELMO^{EID} mutation. **a** and **d** are myoblasts, **b** and **e** are myocytes. The myofibers in panel **c** and **f** are pointed by an arrow. This experiment was repeated twice with myoblasts isolated from different mice Scale bar: 10µm. **g)** Cell migration of primary myoblasts isolated from WT, *Elmo2*$^{EID/EID}$ and *Elmo1*$^{-/-}$*Elmo2*$^{RBD/RBD}$ mice is not affected, since the cells demonstrated similar directionality pattern. **h)** Quantification of the cell speed quantified using the Volocity software. Graph represent the Mean +/- SD of the tracks of myoblasts isolated from 3 different mice per condition. Source data are provided as a Source Data file.

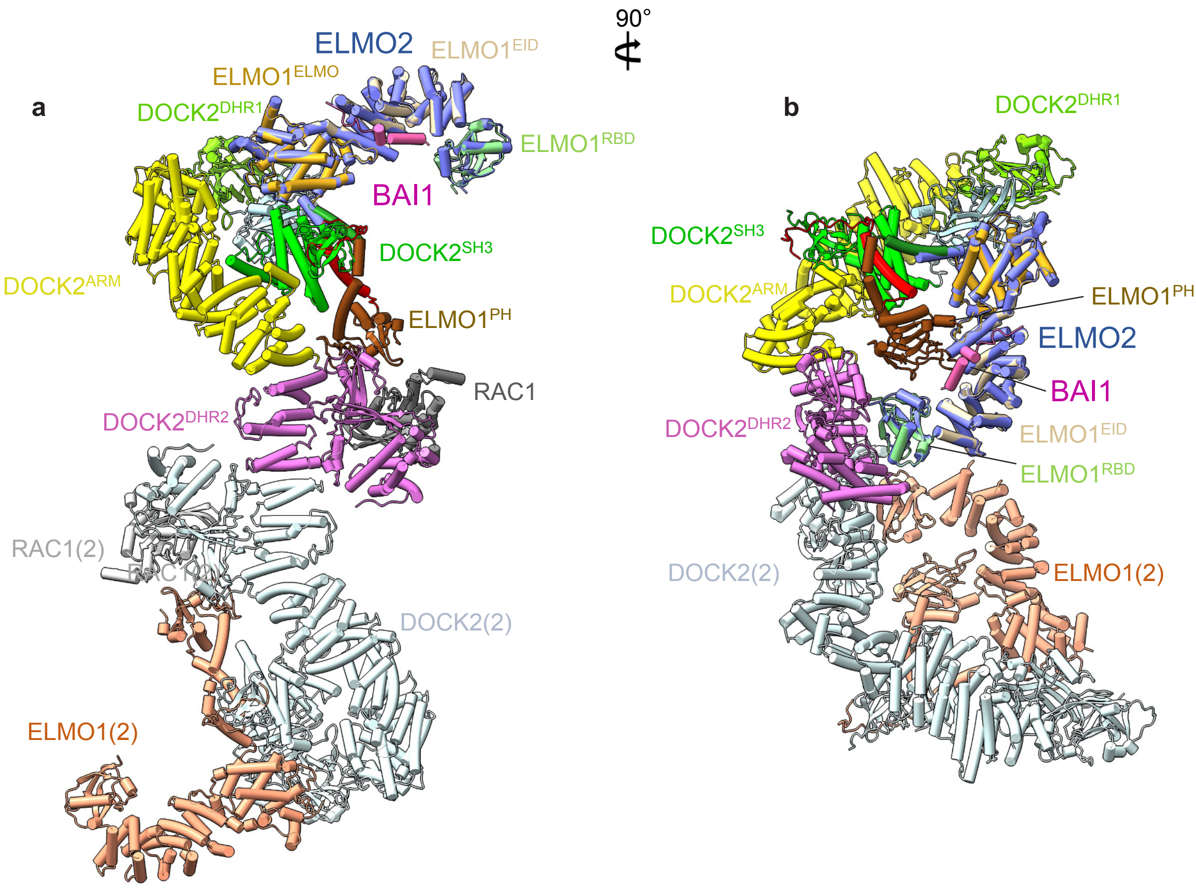

Supplementary Figure 13. Impact of ELMO conformation on BAI receptors-binding site accessibility. a) DOCK2-ELMO1-RAC1 active conformation. ELMO2-BAI1 superimposed onto ELMO1 shows that the BAI receptors-binding site is accessible to BAI binding. **b)** DOCK2-ELMO1 inactive conformation, with ELMO1 adopting the auto-inhibited conformation. ELMO2-BAI1 superimposed onto ELMO1 shows that the BAI receptors-binding site is inaccessible to BAI binding because the residues of the BAI receptors-binding site are sterically blocked by ELMO1^{PH}. BAI1 binding to ELMO1 would therefore stabilize the active state of DOCK2-ELMO1.

Supplemental Table 1 - Mendelian tables of *Elmo2* ^{LacZ}, *Elmo1* $^{-/-}$ *Elmo2* ^{LacZ} and *Meox* ^{CRE} *Elmo2* ^{flox} mice

Elmo2 ^{LacZ} **mice**	**+/+**	**LacZ/+**	**LacZ/LacZ**	**Total of mice**
After weaning	35%	65%	0%	382
E14.5	27%	51%	22%	95
Expected ratio	*25%*	*50%*	*25%*	

Elmo1 $^{-/-}$ ***Elmo2*** ^{LacZ} **mice**	**+/+**	**LacZ/+**	**LacZ/LacZ**	**Total of mice**
After weaning	37%	63%	0%	35
Expected ratio	*25%*	*50%*	*25%*	

Meox ^{CRE} ***Elmo2*** ^{flox} **mice**	**+/+**	**Δ/+**	**Δ/Δ**	**Total of mice**
After weaning	31%	69%	0%	75
Expected ratio	25%	50%	*25%*	

Supplemental Table 2 - Mendelian tables of *Elmo2* ^{flox} and *Elmo1* $^{-/-}$ *Elmo2* ^{flox} mice

Myf5 ^{CRE} ***Elmo2*** ^{flox} **mice**	Cre-			Cre+			**Total of mice**
	**Elmo2** $^{+/+}$	**Elmo2** $^{flox/+}$	**Elmo2** $^{flox/flox}$	**Elmo2** $^{+/+}$	**Elmo2** $^{flox/+}$	**Elmo2** $^{flox/flox}$	
After weaning	13%	28%	10%	6%	24%	10%	79
Expected ratio	*12,5%*	*25%*	*12,5%*	*12,5%*	*25%*	*12,5%*	

Pax3 ^{CRE} ***Elmo2*** ^{flox} **mice**	Cre-			Cre+			**Total of mice**
	**Elmo2** $^{+/+}$	**Elmo2** $^{flox/+}$	**Elmo2** $^{flox/flox}$	**Elmo2** $^{+/+}$	**Elmo2** $^{flox/+}$	**Elmo2** $^{flox/flox}$	
After weaning	27%	25%	13%	10%	18%	7%	83
Expected ratio	*12,5%*	*25%*	*12,5%*	*12,5%*	*25%*	*12,5%*	

Myf5 ^{CRE} ***Elmo1*** $^{-/-}$ ***Elmo2*** ^{flox} **mice**	Cre- Elmo1 $^{-/-}$			Cre+ Elmo1 $^{-/-}$			**Total of mice**
	**Elmo2** $^{+/+}$	**Elmo2** $^{flox/+}$	**Elmo2** $^{flox/flox}$	**Elmo2** $^{+/+}$	**Elmo2** $^{flox/+}$	**Elmo2** $^{flox/flox}$	
After weaning	12%	30%	17%	8%	33%	0%	109
E14.5	12%	19%	19%	10%	24%	16%	58
Expected ratio	*12,5%*	*25%*	*12,5%*	*12,5%*	*25%*	*12,5%*	

Pax3 ^{CRE} ***Elmo1*** $^{-/-}$ ***Elmo2*** ^{flox} **mice**	Cre- Elmo1 $^{-/-}$			Cre+ Elmo1 $^{-/-}$			**Total of mice**
	**Elmo2** $^{+/+}$	**Elmo2** $^{flox/+}$	**Elmo2** $^{flox/flox}$	**Elmo2** $^{+/+}$	**Elmo2** $^{flox/+}$	**Elmo2** $^{flox/flox}$	
After weaning	16%	22%	15%	14	34%	0%	133
E14.5	15%	28%	10%	17%	17%	13%	60
Expected ratio	*12,5%*	*25%*	*12,5%*	*12,5%*	*25%*	*12,5%*	

Supplemental Table 3 - Mendelian tables of Elmo2 knock-in mice

Elmo2 ^{RBD} **mice**	**+/+**	**RBD/+**	**RBD/RBD**	**Total of mice**
After weaning	31%	47%	22%	179
Expected ratio	*25%*	*50%*	*25%*	

Elmo1 $^{-/-}$***Elmo2*** ^{RBD} **mice**	**+/+**	**RBD/+**	**RBD/RBD**	**Total of mice**
After weaning	32%	49%	19%	85
Expected ratio	*25%*	*50%*	*25%*	

Elmo2 ^{EID} **mice**	**+/+**	**EID/+**	**EID/EID**	**Total of mice**
After weaning	29%	56%	15%	411
Expected ratio	*25%*	*50%*	*25%*	

Elmo1 $^{-/-}$***Elmo2*** ^{EID} **mice**	**+/+**	**EID/+**	**EID/EID**	**Total of mice**
After weaning	38%	62%	0%	50
E14.5	22%	60%	18%	45
Expected ratio	*25%*	*50%*	*25%*	

Supplemental Table 4 - Primers used for the experiments

	Forward	Reverse	Experiment
mElmo1	5'-GAGAGCAGAGCAAACACATC-3'	5'-TAATACGACTCACTATAGGGTACAAAAACAAAGCCAACAA-3'	Whole mount *in situ* hybridization
mElmo2	5'-ACATCTGCATCTGGTACTGG-3'	5'-TAATACGACTCACTATAGGGACACATCACTGACAGGAAGC-3'	Whole mount *in situ* hybridization
mElmo3	5'-AGCAGCCACGAAATCACAGA-3'	5'-TAATACGACTCACTATAGGGCTGAGACCTACAATGGTGCC-3'	Whole mount *in situ* hybridization
mElmo1	5'-AAGCAGTGGTGACGGGAAAG-3'	5'-CAAGCACCTCCTTGTTTTGTTTAA-3'	qPCR
mElmo2	5'-ACCGTGGGACAGCTCATCTC-3'	5'-CAATGGCGTAGGTCTGGATCTC-3'	qPCR
mElmo3	5'-GCACTGCCCCAGATCTGAAG-3'	5'-CTTCACGACTTGTATTCTGCAGTCTAC-3'	qPCR
mB2M	5'-CACTGACCGGCCTGTATGC-3'	5'-GGTGGCGTGAGTATACTTGAATTTG-3'	qPCR
B-actine	5'-TGATGGTGGGAATGGGTCAGAA-3'	5'-TCCATGTCGTCCCAGTTGGTAA-3'	qPCR
Mymk	5'-ATCGCTACCAAGAGGCGTT-3'	5'-CACAGCACAGACAAACCAGG-3'	qPCR
Mymx	5'-ACCAGCTTTCATGCCAGAAG-3'	5'-ATGTCTTGGGAGCTCAGTCG-3'	qPCR
Myogenin	5'-TCCCAACCCAGGAGATCATTT-3'	5'-GCAGATTGTGGGCGTCTGTA-3'	qPCR
Myod1	5'-GGCGACTCAGATGCATCCA-3'	5'-CTGTAATCCATCATGCCATCAGA-3'	qPCR
Elmo2^{LacZ}	5'-TGA CGA GGA GCT GCT CTG T-3'	5'-TAGACGCTGCAGTCTGGAGT-3'	Genotyping
Elmo2^{flox}	5'-CTTGTTAACTGTAGAGCACCGTGC-3'	5'-CCGTCTGAGCAGTTGCTCAT-3'	Genotyping
Elmo2^{flox}	5'-CTTGTTAACTGTAGAGCACCGTGC-3'	5'-TGACGAGGAGCTGCTCTGT-3'	PCR - Deletion
Elmo2^{EID}	5'-CTTCTCTGGAGACCTGCACCAAG-3'	5'-TGAGAAGCGCTGGTCAGTAGACAG-3'	Genotyping
Elmo2^{RBD}	5'-TGAAGTGGAAGGCTCTCTGGTC-3'	5'-AACACAAGGCAAGTGCTGGCTG-3'	Genotyping
Pax3^{CRE}<br>Myf5^{CRE}	5'-CGTAGACGCCTGAAGAAGGTCAACCA-3'	5’-CACATTAGAAAACCTGCCAACACC-3’<br>5'-ACGAAGTTATTAGGTCCCTCGAC-3'	Genotyping
Elmo2$^{\Delta}$	5'-CTTGTTAACTGTAGAGCACCGTGC-3'	5’-TGACGAGGAGCTGCTCTGT-3’<br>5’-TAGACGCTGCAGTTCTGGAGT-3’	Genotyping
Elmo1$^{-/-}$	5'-ACCCATATCAGGCTGCCCACATAA-3'<br>5'-GCTTCCTGACGGTGGATGCAATGT-3'	5'-ATGCAATGTGAGCAGCATTCCCTC-3'<br>5'-GGCCGCGTCGACGAAGTTCCTATA-3'	Genotyping
Dysferlin$^{-/-}$	5’-TTCCTCTCTTGTCGGTCTAG-3’<br>5’-GCCTTGATCAGAGTAACTGTC-3’	5'-CTT CAC TGG GAA GTA TGT CG-3'	Genotyping
