# Supplementary material for: Biasing the conformation of ELMO2 reveals that myoblast fusion can be exploited to improve muscle regeneration
Source: Nat Commun. 2022 Nov 18;13:7077. doi: 10.1038/s41467-022-34806-4 (PMC9674853; doi:10.1038/s41467-022-34806-4)
Supplement: Supplementary file 2 — Reporting Summary [file 41467_2022_34806_MOESM2_ESM.pdf]

nature portfolio

Corresponding author(s): Jean-François Côté

Last updated by author(s): Oct 12, 2022

Reporting Summary

Nature Portfolio wishes to improve the reproducibility of the work that we publish. This form provides structure for consistency and transparency in reporting. For further information on Nature Portfolio policies, see our Editorial Policies and the Editorial Policy Checklist.

Statistics

For all statistical analyses, confirm that the following items are present in the figure legend, table legend, main text, or Methods section.

n/a	Confirmed	
[ ]	[x]	The exact sample size (*n*) for each experimental group/condition, given as a discrete number and unit of measurement
[x]	[ ]	A statement on whether measurements were taken from distinct samples or whether the same sample was measured repeatedly
[ ]	[x]	The statistical test(s) used AND whether they are one- or two-sided<br>*Only common tests should be described solely by name; describe more complex techniques in the Methods section.*
[x]	[ ]	A description of all covariates tested
[x]	[ ]	A description of any assumptions or corrections, such as tests of normality and adjustment for multiple comparisons
[ ]	[x]	A full description of the statistical parameters including central tendency (e.g. means) or other basic estimates (e.g. regression coefficient) AND variation (e.g. standard deviation) or associated estimates of uncertainty (e.g. confidence intervals)
[ ]	[x]	For null hypothesis testing, the test statistic (e.g. *F*, *t*, *r*) with confidence intervals, effect sizes, degrees of freedom and *P* value noted<br>*Give P values as exact values whenever suitable.*
[x]	[ ]	For Bayesian analysis, information on the choice of priors and Markov chain Monte Carlo settings
[x]	[ ]	For hierarchical and complex designs, identification of the appropriate level for tests and full reporting of outcomes
[x]	[ ]	Estimates of effect sizes (e.g. Cohen's *d*, Pearson's *r*), indicating how they were calculated

Our web collection on statistics for biologists contains articles on many of the points above.

Software and code

Policy information about availability of computer code

Data collection	Confocal microscopy images were acquired with the Zenblack 2009 software from Carl Zeiss MetaExpress (Version 3.1.097).
Data analysis	For RNA Seq: FASTQC (0.11.5), STAR (2.5.1b), featureCounts (1.4.6), DESeq2(1.34.0), dplyr(1.0.9), ensembldb (2.18.2), ggplot2(3.3.6), ggrepel (0.9.1), GO.db (3.13.0), org.Mm.eg.db (3.13.0), pheatmap (1.0.12), PantherDB (17.0), RColorBrewer (1.1-3), reshape (0.8.9), stringr (1.4.0), svglite (2.1.0). Structural biology analyses: ChimeraX (1.4); NMRView (version 9.2). Isothermal calorimetry: Origin (version 7.0) (Microcal). Statistics: Prism 6 GraphPad (V 6.0). Micosoft Excel (16.9.1). Volocity (version 6.0.0, PerkinElmer Life and Analytical Sciences) and Fiji (version 2.3.0/1.53p) were used to analyze microscopy images.

For manuscripts utilizing custom algorithms or software that are central to the research but not yet described in published literature, software must be made available to editors and reviewers. We strongly encourage code deposition in a community repository (e.g. GitHub). See the Nature Portfolio guidelines for submitting code & software for further information.

Data

Policy information about availability of data

All manuscripts must include a data availability statement. This statement should provide the following information, where applicable:

- Accession codes, unique identifiers, or web links for publicly available datasets
- A description of any restrictions on data availability
- For clinical datasets or third party data, please ensure that the statement adheres to our policy

The raw RNA-seq data presented in Fig. 2h and Supplementary Fig. 9 have been uploaded to the GEO Datasets repository [https://www.ncbi.nlm.nih.gov/gds] and

are available under the following accession number: GSE209546. The remaining data are available within the article, Supplementary Information, or available from the authors upon reasonable request. Fastq file quality was aligned to the publicly available mouse genome (GRCm38). Source data are provided with this paper.

Human research participants

Policy information about studies involving human research participants and Sex and Gender in Research.

Reporting on sex and gender	N/A
Population characteristics	N/A
Recruitment	N/A
Ethics oversight	N/A

Note that full information on the approval of the study protocol must also be provided in the manuscript.

Field-specific reporting

Please select the one below that is the best fit for your research. If you are not sure, read the appropriate sections before making your selection.

- [x] Life sciences
- [ ] Behavioural & social sciences
- [ ] Ecological, evolutionary & environmental sciences

For a reference copy of the document with all sections, see nature.com/documents/nr-reporting-summary-flat.pdf

Life sciences study design

All studies must disclose on these points even when the disclosure is negative.

Sample size	Standard > or = 3 independent experiments were performed for most cases, unless noted in the figure legend. Mouse experiments were designed to have at least 5 animals of each genotype. Experiments using primary cells typically involved isolation of cells from 3 independent mice.
Data exclusions	No data was excluded from this study.
Replication	Experiments were replicated at least 3 times with similar results. The number of time an experiment was replicated is mentioned in the figure legends.
Randomization	Mice selected for study were randomized in the sense that we only relied on genetyping data to identify them. In vitro experiments were randomized since the were done at different times and with different biological samples.
Blinding	Blinding was used in data collection and analysis of RNA-Seq.<br>Blinding was not relevant to the rest of this study because there was no group allocation.

Reporting for specific materials, systems and methods

We require information from authors about some types of materials, experimental systems and methods used in many studies. Here, indicate whether each material, system or method listed is relevant to your study. If you are not sure if a list item applies to your research, read the appropriate section before selecting a response.

Materials & experimental systems

n/a	Involved in the study	
[ ]	[x]	Antibodies
[ ]	[x]	Eukaryotic cell lines
[x]	[ ]	Palaeontology and archaeology
[ ]	[x]	Animals and other organisms
[x]	[ ]	Clinical data
[x]	[ ]	Dual use research of concern

Methods

n/a	Involved in the study	
[x]	[ ]	ChIP-seq
[x]	[ ]	Flow cytometry
[x]	[ ]	MRI-based neuroimaging

Antibodies

Antibodies used	• Monoclonal antibody anti-PAX7, Development Studies Hybridoma Bank (Iowa City, IA), cat. Pax7

• Monoclonal antibody anti-MYOSIN HEAVY CHAIN (MHC - MF20), Development Studies Hybridoma Bank (Iowa City, IA), cat. MF20
• Mouse monoclonal anti-DESMIN, Sigma, cat. D1033
• Mouse monoclonal anti-MYOD, BD Biosciences, cat. 554130, clone 5.8A
• Anti-DYSTROPHIN antibody, Abcam, cat. ab15277
• Anti-phospho HISTONE-H3 (Ser-10) antibody, Cell Signaling, cat. 9701
• Anti-LAMININ DyLight 650 antibody, Novus, cat. NB300-144C
• Anti-ßGalactosidase, Thermo Fisher, cat. A-11132
• Alexa Fluor 488-conjugated-Rat monoclonal anti-F4/80, Biorad, Cat. MCA497A488T, clone A3-1
• Rabbit polyclonal antibody against Elmo was custom generated (Genscript)
• Alexa Fluor 488 chicken anti-mouse IgG, Thermo Fisher, cat. A21200
• Alexa Fluor 488 chicken antirabbit IgG, Thermo Fisher, cat. A21441
• Alexa Fluor 568 goat anti-mouse IgG, Thermo Fisher, cat. A11031
• Alexa Fluor 568 goat anti-rabbit IgG, Thermo Fisher, cat. A11011

Validation

• Monoclonal antibody anti-PAX7: Positive Tested Species Reactivity: Amphibian, Avian, Axolotl, Bovine, Canine, Fish, Goat, Human, Mouse, Ovine, Porcine, Quail, Rat, Turtle, Xenopus, Zebrafish. Recommended Applications: ChIP, FACS, FFPE, Gel Supershift, IF, IHC, IP, WB.
• Monoclonal antibody anti-MYOSIN HEAVY CHAIN (MHC - MF20): Positive Tested Species Reactivity: Amphibian, Avian, Axolotl, Chicken, Fish, Human, Lizard, Mammal, Pig, Snake, Xenopus, Zebrafish. Recommended Applications: ELISA, FACS, FFPE, IF, IHC, IP, WB.
• Mouse monoclonal anti-DESMIN: Reactivity: feline, sheep, bovine, hamster, chicken, viper, rat, rabbit, lizard, mouse, human, goat. Applications: IHC, WB, ICC, ICC localization of intermediate filaments.
• Mouse monoclonal anti-MYOD: Reactivity: QC Testing: Human, Tested in Development: Mouse, Rat, Chicken. Application: WB (routinely tested), IHC-Fr (Tested during development).
• Anti-DYSTROPHIN antibody: Reactivity: Mouse, Human. Tested applications: IHC-Fr, IHC-P.
• Anti-phospho HISTONE-H3 (Ser-10) antibody: Reactivity: Human, Mouse, Rat, Monkey, D. melanogaster. Applications: WB, IHC-P, IF.
• Anti-LAMININ DyLight 650 antibody: Reactivity: Human, Mouse, Rat, Chinese Hamster, Insect, Mammal, Rabbit, Sheep. Reactivity notes: Rabbit, Fruit Bat, Chinese Hamster, and S. mansoni reactivity reported in scientific literature (PMID: 18214989, 31877588, 29251349, and 28114363 respectively). Human, Mouse, Rat, and Sheep reported in multiple pieces of scientific literature. Specificity: Laminin Antibody is pan-specific and reacts well with all Laminin isoforms tested: Laminin-1 (alpha-1, beta-1, and gamma-1) and Laminin-2 (alpha-2, beta-1, and gamma-1). Applications: WB, Flow, ICC/IF, IHC, IHC-Fr, IHC-P, _HC-FrFl. Application notes: This Laminin antibody detects bands at around 440, 220, and 158 kDa in Western Blot. Use in flow cytometry (PMID: 31819166) reported in scientific literature. Use in ICC/IF, IHC, IHC-Frozen, IHC-Paraffin, and Western Blot reported in multiple pieces of scientific literature. Immunostaining is enhanced by antigen retrieval with pepsin, especially paraffin tissue. The observed molecular weight of the protein may vary from the listed predicted molecular weight due to post translational modifications, post translation cleavages, relative charges, and other experimental factors.
• Anti-ßGalactosidase: Reactivity: Bacteria, Fruit fly, Human, Mouse, Rat. Applications: WB, IHC, IHC-P, IHC-Fr, ICC/IF, ELISA, ChIP.
• Alexa Fluor 488-conjugated-Rat monoclonal anti-F4/80: Reactivity: Mouse.Applications: Flow Cytometry.
• Alexa Fluor 488 chicken anti-mouse IgG: Reactivity: Mouse. Applications: IHC, ICC/IF.
• Alexa Fluor 488 chicken antirabbit IgG: Reactivity: Rabbit. Applications: IHC, ICC/IF.
• Alexa Fluor 568 goat anti-mouse IgG: Reactivity: Mouse. Applications: ICC/IF, Flow.
• Alexa Fluor 568 goat anti-rabbit IgG: Reactivity: Rabbit. Applications: IHC-Fr, ICC/IF, Flow.

Eukaryotic cell lines

Policy information about cell lines and Sex and Gender in Research

Cell line source(s)

C2C12 cells were obtained from ATCC.

Authentication

Cells were not authenticated. Instead, the ability of C2C12 to differentiate from progenitors to myotubes when cells switched form proliferation to differentiation was assessed.

Mycoplasma contamination

Cells routinely tested negative for mycoplasma contamination.

Commonly misidentified lines
(See ICLAC register)

The cell line used in this study is not listed in the ICLAC database.

Animals and other research organisms

Policy information about studies involving animals; ARRIVE guidelines recommended for reporting animal research, and Sex and Gender in Research

Laboratory animals

All animals used were on a mixed C57BL6 and 129SV genetic background.

Mouse strains used in the study:
• D2.129S4(B6)-Meox2tm1(cre)Sor/SjJ
• Meox-cre(KI) x Elmo2 H08.B6; Flp
• B6.129S4-Myf5tm3(cre)Sor/J:
• B6.129-Pax3tm1(cre)Joe/J

	• B6.CgPax7 tm1(cre/ERT)Gaka/j<br>• Elmo1 KO.B6<br>• Elmo2 H08.B6.KO<br>• Elmo2 H08.B6 x Cre-Myf5<br>• Elmo2 H08.B6.Flp x Cre-Pax3<br>• Elmo2 EID 2D2xFLP.B6<br>• Elmo2 RBD (L43A) 2B3<br>• Elmo2 L43A 2B3.B6 x Flp.B6<br>• Elmo1 KO.B6 x Elmo2 H08.B6 KO<br>• Elmo1 KO x Elmo2 EID 2D2<br>• Elmo1 Ko.Elmo2 H08.B6 x Cre-Myf5<br>• B6.A-Dysfprmd/GeneJ<br><br>Embryos aged E11.5, E14.5 and E16.5 were sectioned and used for IF staining.<br>Mice aged 2 months were used for cardiotoxin injury experiments.<br>Mice aged 6-8 weeks were used for primary myoblast isolation.<br>Embryos aged E11.5 were used for Whole mount in situ hybridization.<br><br>The mice were kept in ventilated microisolator cages that were autoclaved with bedding and enrichment materials such as Nestlets and wood shavings.<br>All mice are maintained on an irradiated diet (Envigo Teklad Diet 2918) and reverse osmosis water. All cage changes and open-cage procedures were performed under Class 2 biosafety cabinets (BSC). Accelerated hydrogen peroxide was used as a surface disinfectant.<br>The animals are housed in rooms on an automatic dark/light cycle regimen 12-hour light/dark. The Room ambient temperature is set at 21C (+/-2C) and relative humidity is set between 40 and 60%.
Wild animals	Study did not involve wild animals.
Reporting on sex	Sex was not used as a variable in this study.
Field-collected samples	Studies did not involve samples from the field.
Ethics oversight	All animal experiments were approved by he Animal Care Committee of the Institut de Recherches Cliniques de Montreal and complied with the guidelines of the Canadian Council of Animal Care.

Note that full information on the approval of the study protocol must also be provided in the manuscript.
