# Supplementary material for: Biasing the conformation of ELMO2 reveals that myoblast fusion can be exploited to improve muscle regeneration
Source: Nat Commun. 2022 Nov 18;13:7077. doi: 10.1038/s41467-022-34806-4 (PMC9674853; doi:10.1038/s41467-022-34806-4)
Supplement: Supplementary file 4 — Description of Additional Supplementary Files [file 41467_2022_34806_MOESM4_ESM.pdf]

Title: Supplementary dataset 1
Description: RNA-seq gene expression data of Elmo2 wt and Elmo2EID mice
